# Supplementary material for: Pharmacists’ Knowledge, Attitude, and Practice of Medication Therapy Management: A Systematic Review
Source: Healthcare (Basel). 2022 Dec 12;10(12):2513. doi: 10.3390/healthcare10122513 (PMC9778396; doi:10.3390/healthcare10122513)
Supplement: Supplementary file 1 [file healthcare-10-02513-s001.zip › healthcare-2053702-supplementary/Supplementary materials_healthcare 2053702_221126/PRESS guideline_healthcare-2053702_221126.pdf]

*PRESS Guideline* **— Search Submission & Peer Review Assessment**

# SEARCH SUBMISSION: THIS SECTION TO BE FILLED IN BY THE SEARCHER

| | |
|---|---|
| Searcher: Farida Rendrayani | Email: farida21004@mail.unpad.ac.id |
| Date submitted: 1st September, 2022 | Date requested by: 5th September, 2022 |

**Systematic Review Title:**

Pharmacists' knowledge, attitude, and practice of medication therapy management: A systematic review

This search strategy is …

| | |
|---|---|
| √ | My PRIMARY (core) database strategy — First time submitting a strategy for search question and database |
| | My PRIMARY (core) strategy — Follow-up review NOT the first time submitting a strategy for search question and database. If this is a response to peer review, itemize the changes made to the review suggestions |
| | SECONDARY search strategy— First time submitting a strategy for search question and database |
| | SECONDARY search strategy — NOT the first time submitting a strategy for search question and database. If this is a response to peer review, itemize the changes made to the review suggestions |

**Database**

(i.e., MEDLINE,CINAHL…): *[mandatory]*

MEDLINE, Academic Search Complete

**Interface**

(i.e., Ovid, EBSCO…): *[mandatory]*

PubMed, EBSCO

**Research Question**

(Describe the purpose of the search) *[mandatory]*

This study was conducted to systematically review knowledge, attitude, and practice studies on medication therapy management provision among pharmacists worldwide. It focuses on obtaining a complete understanding of the pharmacists' point of view.

**PICO Format**

Instead of using PICO format, we set the search terms based on the key concept.

| | |
|---|---|
| **Concept 1** | Medication therapy management |
| **Concept 2** | Pharmacist |
| **Concept 3** | Knowledge, attitude, practice |

**Inclusion Criteria**

(List criteria such as age groups, study designs, etc., to be included) *[optional]*

The inclusion criteria were original research, quantitative survey, and studies that assessed pharmacists' knowledge, attitude, or practice toward MTM provision.

**Exclusion Criteria**

(List criteria such as study designs, date limits, etc., to be excluded) *[optional]*

We excluded abstracts from conference proceedings, case reports, commentaries, editorials, or study protocols.

**Was a search filter applied?**

Yes ☑ No ■

**If YES, which one(s) (e.g., Cochrane RCT filter, PubMed Clinical Queries filter)? Provide the source if this is a published filter.** *[mandatory if YES to previous question — textbox]*

Publication date

Other notes or comments you feel would be useful for the peer reviewer? ***[optional]***

The publication date was set from 2008 and beyond to avoid irrelevant results concerning medication management because "medication therapy management" was introduced as a MeSH term in 2008. A hand search was conducted by bibliographic "snowballing" and a particular search to supplement the database search. We included the relevant studies published before 2008 in the hand search.

Please copy and paste your search strategy here, exactly as run, including the number of hits per line. ***[mandatory]***

### A. MEDLINE on PubMed

| Search terms | | Number of hits |
|---|---|---|
| 1. | "medication therapy management"[MeSH Terms] OR "medication therapy management"[Title/Abstract] OR "MTM"[Title/Abstract] | 4,292 |
| 2. | “medication”[Title/Abstract] AND “therapy”[Title/Abstract] AND “management”[Title/Abstract] | 11,303 |
| 3. | "drug therapy management"[Title/Abstract] OR "drug therapy service*"[Title/Abstract] OR "medication management service*"[Title/Abstract] | 324 |
| 4. | "medication therapy review*"[Title/Abstract] OR "medicine use review*"[Title/Abstract] OR "personal medication record*"[Title/Abstract] OR "medication action plan*"[Title/Abstract] | 76 |
| 5. | 1 OR 2 OR 3 OR 4 | 14,530 |
| 6. | "pharmacists"[MeSH Terms] OR "pharmacy technicians"[MeSH Terms] OR "pharmacist*"[Title/Abstract] OR "pharmacy technician*"[Title/Abstract] | 45,168 |
| 7. | "knowledge"[MeSH Terms] OR "knowledge"[Title/Abstract] | 856,394 |
| 8. | "attitude"[MeSH Terms] OR "attitude*"[Title/Abstract] | 711,867 |
| 9. | 7 AND 8 AND “practice*”[Title/Abstract] | 38,923 |
| 10. | "health knowledge, attitudes, practice"[MeSH Terms] OR "attitude of health personnel"[MeSH Terms] OR "KAP"[Title/Abstract] | 278,032 |
| 11. | 9 OR 10 | 289,959 |
| 12. | 5 AND 6 AND 11 | 199 |
| 13. | 12 AND (("2008/01/01"[Date - Publication] : "2022/08/31"[Date - Publication])) | 184 |

### B. Academic Search Complete on EBSCO

| Search terms | | Number of hits |
|---|---|---|
| 1. | medication therapy management<br>(find all my search terms, apply equivalent subjects) | 14.551 |
| 2. | drug therapy service*<br>(find all my search terms, apply equivalent subjects) | 31.847 |
| 3. | P1 OR P2<br>(boolean/phrase, apply equivalent subjects) | 44.233 |

| | | |
|---|---|---|
| 4. | pharmacist*<br>(find all my search terms, apply equivalent subjects) | 35.493 |
| 5. | pharmacy technician*<br>(find all my search terms, apply equivalent subjects) | 766 |
| 6. | P4 OR P5<br>(boolean/phrase, apply equivalent subjects) | 35.757 |
| 7. | knowledge, attitude* and practice*<br>(find all my search terms, apply equivalent subjects) | 22.664 |
| 8. | P3 AND P6 AND P7<br>(boolean/phrase, apply equivalent subjects) | 47 |
| 9. | P8<br>Limiters - Published Date: until 20220131 | 47 |

## PEER REVIEW ASSESSMENT: THIS SECTION TO BE FILLED IN BY THE REVIEWER

| | | |
|---|---|---|
| Reviewer: Sofa Dewi Alfian | Email: sofa.alfian@unpad.ac.id | Date completed: 5th September, 2022 |

### 1. TRANSLATION

| | |
|---|---|
| A ---No revisions | ☑ |
| B --- Revision(s) suggested | ☐ |
| C --- Revision(s) required | ☐ |

If "B" or "C," please provide an explanation or example:

### 2. BOOLEAN AND PROXIMITY OPERATORS

| | |
|---|---|
| A ---No revisions | ☑ |
| B --- Revision(s) suggested | ☐ |
| C --- Revision(s) required | ☐ |

If "B" or "C," please provide an explanation or example:

### 3. SUBJECT HEADINGS

| | |
|---|---|
| A ---No revisions | ☑ |
| B --- Revision(s) suggested | ☐ |
| C --- Revision(s) required | ☐ |

If "B" or "C," please provide an explanation or example:

**4. TEXT WORD SEARCHING**

| | |
|---|---|
| A ---No revisions | ☑ |
| B --- Revision(s)suggested | ☐ |
| C --- Revision(s) required | ☐ |

If "B" or "C," please provide an explanation or example:

**5. SPELLING, SYNTAX, AND LINE NUMBERS**

| | |
|---|---|
| A ---No revisions | ☑ |
| B --- Revision(s)suggested | ☐ |
| C --- Revision(s) required | ☐ |

If "B" or "C," please provide an explanation or example:

**6. LIMITS AND FILTERS**

| | |
|---|---|
| A ---No revisions | ☑ |
| B --- Revision(s) suggested | ☐ |
| C --- Revision(s) required | ☐ |

If "B" or "C," please provide an explanation or example:

OVERALL EVALUATION (Note: If one or more "revision required" is noted above, the response below must be "revisions required".)

| | |
|---|---|
| A ---No revisions | ☑ |
| B --- Revision(s) suggested | ☐ |
| C --- Revision(s) required | ☐ |

Additional comments:
