# Supplementary material for: Pharmacists’ Knowledge, Attitude, and Practice of Medication Therapy Management: A Systematic Review
Source: Healthcare (Basel). 2022 Dec 12;10(12):2513. doi: 10.3390/healthcare10122513 (PMC9778396; doi:10.3390/healthcare10122513)
Supplement: Supplementary file 1 [file healthcare-10-02513-s001.zip › healthcare-2053702-supplementary/Supplementary materials_healthcare 2053702_221126/Supplementary Materials_healthcare-2053702_221126.pdf]

Table S1. PRISMA 2020 main checklist.

Topic	No.	Item	Location where item is reported
**TITLE**			
**Title**	1	Identify the report as a systematic review.	page 1
**ABSTRACT**			
**Abstract**	2	See the PRISMA 2020 for Abstracts checklist	
**INTRODUCTION**			
**Rationale**	3	Describe the rationale for the review in the context of existing knowledge.	page 2
**Objectives**	4	Provide an explicit statement of the objective(s) or question(s) the review addresses.	page 2
**METHODS**			
**Eligibility criteria**	5	Specify the inclusion and exclusion criteria for the review and how studies were grouped for the syntheses.	page 2 - 3
**Information sources**	6	Specify all databases, registers, websites, organizations, reference lists and other sources searched or consulted to identify studies. Specify the date when each source was last searched or consulted.	page 2
**Search strategy**	7	Present the full search strategies for all databases, registers and websites, including any filters and limits used.	Supplementary materials, Table S3 and S4
**Selection process**	8	Specify the methods used to decide whether a study met the inclusion criteria of the review, including how many reviewers screened each record and each report retrieved, whether they worked independently, and if applicable, details of automation tools used in the process.	page 2 - 3
**Data collection process**	9	Specify the methods used to collect data from reports, including how many reviewers collected data from each report, whether they worked independently, any processes for obtaining or confirming data from study investigators, and if applicable, details of automation tools used in the process.	page 3

Topic	No.	Item	Location where item is reported
**Data items**	10a	List and define all outcomes for which data were sought. Specify whether all results that were compatible with each outcome domain in each study were sought (for example, all measures, time points, analyses), and if not, the methods used to decide which results to collect.	page 3
	10b	List and define all other variables for which data were sought (such as participant and intervention characteristics, funding sources). Describe any assumptions made about any missing or unclear information.	page 3
**Study risk of bias assessment**	11	Specify the methods used to assess risk of bias in the included studies, including details of the tool(s) used, how many reviewers assessed each study and whether they worked independently, and if applicable, details of automation tools used in the process.	Page 3
**Effect measures**	12	Specify for each outcome the effect measure(s) (such as risk ratio, mean difference) used in the synthesis or presentation of results.	NA
**Synthesis methods**	13a	Describe the processes used to decide which studies were eligible for each synthesis (for example, tabulating the study intervention characteristics and comparing against the planned groups for each synthesis (item 5)).	NA
	13b	Describe any methods required to prepare the data for presentation or synthesis, such as handling of missing summary statistics, or data conversions.	NA
	13c	Describe any methods used to tabulate or visually display results of individual studies and syntheses.	NA
	13d	Describe any methods used to synthesize results and provide a rationale for the choice(s). If meta-analysis was performed, describe the model(s), method(s) to identify the presence and extent of statistical heterogeneity, and software package(s) used.	NA
	13e	Describe any methods used to explore possible causes of heterogeneity among study results (such as subgroup analysis, meta-regression).	NA
	13f	Describe any sensitivity analyses conducted to assess robustness of the synthesized results.	NA

Topic	No.	Item	Location where item is reported
**Reporting bias assessment**	14	Describe any methods used to assess risk of bias due to missing results in a synthesis (arising from reporting biases).	NA
**Certainty assessment**	15	Describe any methods used to assess certainty (or confidence) in the body of evidence for an outcome.	NA
**RESULTS**			
**Study selection**	16a	Describe the results of the search and selection process, from the number of records identified in the search to the number of studies included in the review, ideally using a flow diagram.	page 3
	16b	Cite studies that might appear to meet the inclusion criteria, but which were excluded, and explain why they were excluded.	page 3
**Study characteristics**	17	Cite each included study and present its characteristics.	page 3 - 4
**Risk of bias in studies**	18	Present assessments of risk of bias for each included study.	page 17, Supplementary materials Table S5
**Results of individual studies**	19	For all outcomes, present, for each study: (a) summary statistics for each group (where appropriate) and (b) an effect estimation and its precision (confidence/credible interval), ideally using structured tables or plots.	page 6 – 7, page 9 – 10, page 13 - 16
**Results of syntheses**	20a	For each synthesis, briefly summarize the characteristics and risk of bias among contributing studies.	NA
	20b	Present results of all statistical syntheses conducted. If meta-analysis was done, present for each the summary estimate and its precision (confidence/credible interval) and measures of statistical heterogeneity. If comparing groups, describe the direction of the effect.	NA
	20c	Present results of all investigations of possible causes of heterogeneity among study results.	NA
	20d	Present results of all sensitivity analyses conducted to assess the robustness of the synthesized results.	NA
**Reporting biases**	21	Present assessments of risk of bias due to missing results (arising from reporting biases) for each synthesis assessed.	NA

Topic	No.	Item	Location where item is reported
**Certainty of evidence**	22	Present assessments of certainty (or confidence) in the body of evidence for each outcome assessed.	NA
**DISCUSSION**			
**Discussion**	23a	Provide a general interpretation of the results in the context of other evidence.	page 17 - 19
	23b	Discuss any limitations of the evidence included in the review.	page 19
	23c	Discuss any limitations of the review processes used.	page 20
	23d	Discuss implications of the results for practice, policy, and future research.	page 20
**OTHER INFORMATION**			
**Registration and protocol**	24a	Provide registration information for the review, including register name and registration number, or state that the review was not registered.	none
	24b	Indicate where the review protocol can be accessed, or state that a protocol was not prepared.	none
	24c	Describe and explain any amendments to information provided at registration or in the protocol.	none
**Support**	25	Describe sources of financial or non-financial support for the review, and the role of the funders or sponsors in the review.	page 20
**Competing interests**	26	Declare any competing interests of review authors.	page 20
**Availability of data, code and other materials**	27	Report which of the following are publicly available and where they can be found: template data collection forms; data extracted from included studies; data used for all analyses; analytic code; any other materials used in the review.	page 20

NA = not applicable

Table S2. PRISMA abstract checklist.

Topic	No.	Item	Reported?
**TITLE**			
**Title**	1	Identify the report as a systematic review.	Yes
**BACKGROUND**			
**Objectives**	2	Provide an explicit statement of the main objective(s) or question(s) the review addresses.	Yes
**METHODS**			
**Eligibility criteria**	3	Specify the inclusion and exclusion criteria for the review.	Yes
**Information sources**	4	Specify the information sources (databases, registers) used to identify studies and the date when each was last searched.	Yes
**Risk of bias**	5	Specify the methods used to assess risk of bias in the included studies.	No
**Synthesis of results**	6	Specify the methods used to present and synthesize results.	Yes
**RESULTS**			
**Included studies**	7	Give the total number of included studies and participants and summarize relevant characteristics of studies.	Yes
**Synthesis of results**	8	Present results for main outcomes, preferably indicating the number of included studies and participants for each. If meta-analysis was done, report the summary estimate and confidence/credible interval. If comparing groups, indicate the direction of the effect (which group is favored).	Yes
**DISCUSSION**			
**Limitations of evidence**	9	Provide a brief summary of the limitations of the evidence included in the review (study risk of bias, inconsistency and imprecision).	Yes
**Interpretation**	10	Provide a general interpretation of the results and important implications.	Yes
**OTHER**			
**Funding**	11	Specify the primary source of funding for the review.	No
**Registration**	12	Provide the register name and registration number.	No

Table S3. Search strategy of MEDLINE on PubMed.

	Search terms	Number of hits
1.	"medication therapy management"[MeSH Terms] OR "medication therapy management"[Title/Abstract] OR "MTM"[Title/Abstract]	4,292
2.	“medication”[Title/Abstract] AND “therapy”[Title/Abstract] AND “management”[Title/Abstract]	11,303
3.	"drug therapy management"[Title/Abstract] OR "drug therapy service*"[Title/Abstract] OR "medication management service*"[Title/Abstract]	324
4.	"medication therapy review*"[Title/Abstract] OR "medicine use review*"[Title/Abstract] OR "personal medication record*"[Title/Abstract] OR "medication action plan*"[Title/Abstract]	76
5.	1 OR 2 OR 3 OR 4	14,530
6.	"pharmacists"[MeSH Terms] OR "pharmacy technicians"[MeSH Terms] OR "pharmacist*"[Title/Abstract] OR "pharmacy technician*"[Title/Abstract]	45,168
7.	"knowledge"[MeSH Terms] OR "knowledge"[Title/Abstract]	856,394
8.	"attitude"[MeSH Terms] OR "attitude*"[Title/Abstract]	711,867
9.	7 AND 8 AND “practice*”[Title/Abstract]	38,923
10.	"health knowledge, attitudes, practice"[MeSH Terms] OR "attitude of health personnel"[MeSH Terms] OR "KAP"[Title/Abstract]	278,032
11.	9 OR 10	289,959
12.	5 AND 6 AND 11	199
13.	12 AND (("2008/01/01"[Date - Publication] : "2022/08/31"[Date - Publication]))	184

Table S4. Search strategy of Academic Search Complete on EBSCO.

	Search terms	Number of hits
1.	medication therapy management<br>(find all my search terms, apply equivalent subjects)	14.551
2.	drug therapy service*<br>(find all my search terms, apply equivalent subjects)	31.847
3.	P1 OR P2<br>(boolean/phrase, apply equivalent subjects)	44.233
4.	pharmacist*<br>(find all my search terms, apply equivalent subjects)	35.493
5.	pharmacy technician*<br>(find all my search terms, apply equivalent subjects)	766
6.	P4 OR P5<br>(boolean/phrase, apply equivalent subjects)	35.757
7.	knowledge, attitude* and practice*<br>(find all my search terms, apply equivalent subjects)	22.664
8.	P3 AND P6 AND P7<br>(boolean/phrase, apply equivalent subjects)	47
9.	P8<br>Limiters - Published Date: until 20220131	47

Table S5. Checklist for Reporting of Survey Studies (CROSS) for all included studies in the review.

Section / topic	Item	Item description	References																
			Alshehri et al., 2022 [32]	Jarab et al., 2022 [33]	Akonoghrere et al., 2020 [34]	Akonoghrere et al., 2020 [35]	Al-Tameemi and Sarriff, 2019 [36]	Domiati et al., 2018 [37]	Brown et al., 2018 [38]	Battaglia et al., 2012 [39]	Shah and Chawla, 2011 [40]	Law et al., 2009 [41]	MacIntosh et al., 2009 [42]	Herbert et al., 2006 [43]	Blake and Madhavan, 2010 [44]	Lounsbery et al., 2009 [45]	Bright et al., 2009 [46]	Blake et al., 2009 [47]	Moczygemba et al., 2008 [48]
		**Title and abstract**																	
Title and abstract	1a	State the word "survey" along with a commonly used term in title or abstract to introduce the study's design.	R	R	R	R	R	R	R	R	R	R	R	R	R	R	R	R	R
	1b	Provide an informative summary in the abstract, covering background, objectives, methods, findings/results, interpretation/discussion, and conclusions.	R	R	R	R	R	R	R	R	R	R	R	R	R	R	R	R	R
		**Introduction**																	
Background	2	Provide a background about the rationale of study, what has been previously done, and why this survey is needed.	R	R	P	P	P	R	R	R	R	P	R	R	R	P	R	R	p
Purpose/aim	3	Identify specific purposes, aims, goals, or objectives of the study.	R	R	R	R	R	R	R	R	R	R	R	R	R	R	R	R	R
		**Methods**																	
Study design	4	Specify the study design in the methods section with a commonly used term (e.g., cross-sectional or longitudinal).	R	R	R	R	R	R	N	N	R	N	R	R	N	R	N	N	R
Data collection methods	5a	Describe the questionnaire (e.g., number of sections, number of questions, number and names of instruments used).	R	R	R	R	R	R	R	R	R	R	R	R	R	R	R	R	R
	5b	Describe all questionnaire instruments that were used in the survey to measure particular concepts. Report target population, reported validity and reliability information, scoring/classification procedure, and reference links (if any).	R	R	R	R	R	R	P	P	R	R	P	R	R	P	P	R	R
	5c	Provide information on pretesting of the questionnaire, if performed (in the article or in an online supplement). Report the method of pretesting, number of times questionnaire was pre-tested, number and demographics of participants used for pretesting, and the level of similarity of demographics between pre-testing participants and sample population.	P	P	P	P	P	R	N	N	N	N	P	R	P	R	R	R	R
	5d	Questionnaire, if possible, should be fully provided (in the article, or as appendices or as an online supplement).	R	R	R	R	R	R	N	R	R	P	R	R	R	P	R	N	R
Sample characteristics	6a	Describe the study population (i.e., background, locations, eligibility criteria for participant inclusion in survey, exclusion criteria).	R	R	R	R	R	R	R	R	R	R	R	R	R	R	R	R	R

	6b	Describe the sampling techniques used (e.g., single stage or multistage sampling, simple random sampling, stratified sampling, cluster sampling, convenience sampling). Specify the locations of sample participants whenever clustered sampling was applied.	R	R	R	R	R	R	R	R	R	R	R	R	R	R	R	R	R
	6c	Provide information on sample size, along with details of sample size calculation.	N	N	R	R	R	R	R	R	R	R	R	R	R	R	R	R	R
	6d	Describe how representative the sample is of the study population (or target population if possible), particularly for population-based surveys.	N	N	N	N	NA	R	NA	NA	NA	N	NA	R	R	R	NA	R	R
Survey administration	7a	Provide information on modes of questionnaire administration, including the type and number of contacts, the location where the survey was conducted (e.g., outpatient room or by use of online tools, such as SurveyMonkey).	R	R	R	R	R	R	R	R	R	R	R	R	R	R	R	R	R
	7b	Provide information of survey’s time frame, such as periods of recruitment, exposure, and follow-up days.	R	N	N	N	R	R	R	R	R	R	R	R	R	R	R	R	R
	7c	Provide information on the entry process: –>For non-web-based surveys, provide approaches to minimize human error in data entry. –>For web-based surveys, provide approaches to prevent “multiple participation” of participants.	N	N	N	N	N	R	R	R	N	N	R	N	N	N	N	N	N
Study preparation	8	Describe any preparation process before conducting the survey (e.g., interviewers’ training process, advertising the survey).	R	R	N	N	N	R	R	R	N	R	R	R	R	R	R	R	R
Ethical considerations	9a	Provide information on ethical approval for the survey if obtained, including informed consent, institutional review board [IRB] approval, Helsinki declaration, and good clinical practice [GCP] declaration (as appropriate).	P	R	N	N	R	R	R	R	R	R	R	R	R	R	R	R	R
	9b	Provide information about survey anonymity and confidentiality and describe what mechanisms were used to protect unauthorized access.	P	N	N	N	P	R	NA	NA	R	R	R	R	R	R	R	N	R
Statistical analysis	10a	Describe statistical methods and analytical approach. Report the statistical software that was used for data analysis.	R	R	R	R	R	R	R	R	R	R	R	R	R	R	R	R	R
	10b	Report any modification of variables used in the analysis, along with reference (if available).	NA	NA	NA	NA	NA	NA	NA	NA	NA	NA	NA	NA	NA	NA	NA	NA	NA

	10c	Report details about how missing data was handled. Include rate of missing items, missing data mechanism (i.e., missing completely at random [MCAR], missing at random [MAR] or missing not at random [MNAR]) and methods used to deal with missing data (e.g., multiple imputation).	P	N	N	N	N	NA	NA	NA	N	N	R	NA	P	N	N	N	N
	10d	State how non-response error was addressed.	NA	NA	NA	NA	N	N	NA	NA	N	NA	NA	NA	R	N	R	R	N
	10e	For longitudinal surveys, state how loss to follow-up was addressed.	NA	NA	NA	NA	NA	NA	N	N	NA	NA	NA	NA	NA	NA	NA	NA	NA
	10f	Indicate whether any methods such as weighting of items or propensity scores have been used to adjust for non-representativeness of the sample.	N	N	N	N	NA	NA	NA	NA	NA	N	NA	N	N	N	N	N	N
	10g	Describe any sensitivity analysis conducted.	NA	NA	NA	NA	NA	NA	NA	NA	NA	NA	NA	NA	NA	NA	NA	NA	NA
**Results**																			
Respondent characteristics	11a	Report numbers of individuals at each stage of the study. Consider using a flow diagram, if possible.	NA	NA	NA	NA	NA	NA	NA	NA	NA	NA	NA	NA	NA	NA	NA	NA	NA
	11b	Provide reasons for non-participation at each stage, if possible.	NA	NA	NA	NA	NA	NA	NA	NA	NA	NA	NA	NA	NA	NA	NA	NA	NA
	11c	Report response rate, present the definition of response rate or the formula used to calculate response rate.	R	R	R	R	R	R	R	R	R	R	R	R	R	R	R	R	R
	11d	Provide information to define how unique visitors are determined. Report number of unique visitors along with relevant proportions (e.g., view proportion, participation proportion, completion proportion).	NA	NA	NA	NA	NA	NA	NA	NA	NA	NA	NA	NA	NA	NA	NA	NA	NA
Descriptive results	12	Provide characteristics of study participants, as well as information on potential confounders and assessed outcomes.	R	R	R	R	R	R	R	R	R	R	N	R	R	R	R	N	R
Main findings	13a	Give unadjusted estimates and, if applicable, confounder-adjusted estimates along with 95% confidence intervals and p-values.	R	R	R	R	R	R	R	R	R	N	R	R	R	R	N	NA	R
	13b	For multivariable analysis, provide information on the model building process, model fit statistics, and model assumptions (as appropriate).	N	R	NA	NA	NA	R	NA	NA	NA	NA	NA	R	NA	R	NA	NA	N
	13c	Provide details about any sensitivity analysis performed. If there are considerable amount of missing data,	NA	NA	NA	NA	NA	NA	NA	NA	NA	NA	NA	NA	NA	NA	NA	NA	NA

		report sensitivity analyses comparing the results of complete cases with that of the imputed dataset (if possible).																	
**Discussion**																			
Limitations	14	Discuss the limitations of the study, considering sources of potential biases and imprecisions, such as non-representativeness of sample, study design, important uncontrolled confounders.	R	R	N	N	R	R	R	R	R	R	R	R	R	R	R	R	R
Interpretations	15	Give a cautious overall interpretation of results, based on potential biases and imprecisions and suggest areas for future research.	R	R	P	P	R	P	R	R	R	R	R	R	R	R	R	R	P
Generalizability	16	Discuss the external validity of the results.	N	N	N	N	NA	R	NA	NA	NA	N	NA	R	R	R	N	R	R
**Other sections**																			
Role of funding source	17	State whether any funding organization has had any roles in the survey's design, implementation, and analysis.	R	R	N	N	R	R	R	R	R	N	R	N	R	R	R	N	N
Conflict of interest	18	Declare any potential conflict of interest.	R	R	N	R	R	R	R	N	R	N	R	N	N	R	N	N	R
Acknowledge-ments	19	Provide names of organizations/persons that are acknowledged along with their contribution to the research.	NA	NA	NA	NA	R	R	R	R	NA	R	R	N	R	R	R	N	N
**Reporting quality based on author judgment***			**M**	**M**	**M**	**M**	**H**	**H**	**H**	**H**	**H**	**M**	**H**	**H**	**H**	**H**	**H**	**M**	**H**

R = reported, P = partially reported, N = not reported, NA: not applicable, M = moderate, H = high.
*Studies were assigned 1 point for reporting the item, 0.5 for partially reporting, and 0 for not reporting. Percentages were calculated after the exclusion of "not applicable" items. Studies were categorized as 'high quality' if the reporting quality ≥ 75%, 'moderate' if the quality ranged between 50% and 75%, and 'low' if less than 50%.
